# Role of Lysogenic Phages in the Dissemination of Antibiotic Resistance Genes Applied in the Food Chain

**DOI:** 10.3390/foods14071082

**Published:** 2025-03-21

**Authors:** Rafael Dorighello Cadamuro, Mariana Alves Elois, Giulia Von Tönnemann Pilati, Beatriz Pereira Savi, Leonardo Pessi, Yasmin Ferreira Souza Hoffmann Jempierre, David Rodríguez-Lázaro, Gislaine Fongaro

**Affiliations:** 1 Department of Microbiology, Immunology and Parasitology, Federal University of Santa Catarina, Florianópolis 88040-900, SC, Brazil; malves@ubu.es (M.A.E.); giuliavpilati@gmail.com (G.V.T.P.); beasavis2@gmail.com (B.P.S.); pessileonardo@gmail.com (L.P.); yasminfshoffmann@gmail.com (Y.F.S.H.J.); gislaine.fongaro@gmail.com (G.F.); 2 Microbiology Division, Faculty of Sciences, University of Burgos, 09001 Burgos, Spain; 3 Research Centre for Emerging Pathogens and Global Health, University of Burgos, 09001 Burgos, Spain

**Keywords:** bacteriophages, antibiotic resistance genes (ARGs), food safety, lateral transduction, horizontal gene transfer, lysogeny

## Abstract

Bacteriophages, first discovered in 1915, have re-emerged as critical players in microbial ecosystems, particularly in food production. Their ability to lysogenize bacterial hosts raises concerns about their role in the horizontal transfer of antibiotic resistance genes (ARGs) and virulence factors, contributing to the global challenge of antimicrobial resistance. Key studies reveal that ARG-carrying phages are prevalent across various stages of the food chain, including soil, vegetables, meat, dairy, and wastewater associated with food production. These findings demonstrate the potential for lysogenic phages to act as vectors for resistance gene dissemination, posing risks to public health. The review also explores emerging genetic elements, such as phage-inducible chromosomal islands and gene transfer agents, that further enhance the mobility of resistance and virulence genes. Advancements in metagenomic tools have improved our understanding of phage-mediated gene transfer, but significant knowledge gaps remain. Future research should aim to quantify these processes in real-world settings and develop strategies to mitigate the risks associated with lysogenic phages in food systems.

## 1. Introduction

Bacteria are prokaryotes cells that can inhabit and colonize distinct environments, creating communities. Those communities can be influenced by factors such as composition of species, pH, temperature, and others. Among those factors exist the bacteriophages, viruses that replicate inside bacteria.

The discovery of phages was first related in 1896, when British bacteriologist Ernest Hanbury noticed antibacterial activity in the waters of the Ganga and Yamuna rivers in India. He noted that a filterable and heat-sensitive substance could inhibit bacterial infections, although the agent responsible for this phenomenon was not identified at the time [1,2]. Years later, the bacteriology professor Frederick William Twort and the microbiologist Félix d’Hérelle independently described agents that inhibited bacterial growth. In this way, the discovery of bacteriophages was made by both scientists independently [3].

Today, phages are recognized as the most abundant and diverse biological entities on Earth, with an estimated 10^31^ particles in the biosphere. They can be found in all ecosystems, from extreme environments such as hydrothermal areas and deserts to polar regions and hypersaline habitats [4,5,6].

The marine environment is one of the ecosystems with the highest natural abundance of bacteriophages and other viruses, averaging approximately 10 million viral particles per milliliter [7]. Similarly, phage densities typically range from 10^8^ to 10^9^ particles per gram of soil in both forest floors and agricultural soils [8,9]. Considering genetic material, phages can have DNA or RNA, which may be double-stranded or single-stranded. The capsid is a protein structure that covers and protects genetic material. Some phages also have a structure called a viral envelope, composed of lipid membranes from the host, and containing recognition proteins to initiate the attachment step on the bacterial surface [10].

The taxonomic classification of viruses is realized by the International Committee on Taxonomy of Viruses (ICTV). However, during the years the classification of phages utilized characteristics such as morphology, genome type, and host range. Those characteristics eventually construct polyphyletic clusters, which do not correlate with their evolutionary history. In 2022, ICTV restructured phage taxonomy removing polyphyletic groups, introducing a new order, Crassvirales, which comprise double-stranded DNA-tailed phages with genomes ranging from 83 to 106 kbp. This reformulation established 21 new families, reorganized existing families, and introduced additional subfamilies [11,12].

In the year 2024, the National Center for Biotechnology Information (NCBI) database contained 17,708 complete phage genomes, classified into 4 orders, 47 families, 1199 genera, and 3601 species [9,12].

Phages are categorized into two groups with different replicative cycles: lytic and lysogenic. In the lytic cycle, phages infect a bacterial host, hijack enzymes to force the host to produce new phage particles, which contribute to the lyse of the host cell, releasing progeny. The lysogenic cycle involves the integration of phage DNA into the host’s chromosome by integrases enzyme, where it remains dormant as a prophage until stimulated by environmental or cellular stress to transition into the lytic cycle [9].

In the last years, research has shown the importance of phage-mediated gene transfer mechanisms, such as lateral transduction, which operate at higher frequencies compared to other transduction forms. This process contributes to the emergence of multidrug-resistant pathogens in different matrices, such as the environment, animals, industry, and humans. Since all these matrices are connected, an increase in one can influence another. In this way, the selection of multidrug-resistant bacteria in the environment can impact animal health, animal production, and even human health. This review aims to assess the risks posed by lysogenic bacteriophages in food production systems, especially regarding their role in the HGT of ARGs and virulence factors. By focusing on emerging mechanisms like lateral transduction and integrating recent discoveries, this study aims to detect resistance genes in both phage fragments and bacteria in food matrices, while also highlighting critical gaps in the understanding of antimicrobial resistance dissemination in this context.

## 2. Horizontal Gene Transfer

It is well established that bacteriophages play a crucial role in bacterial evolution and pathogenesis by facilitating the transfer of genetic material between different bacterial species [13,14,15,16].

It is possible to quantify the number of viruses generated after the replication process in bacterial cells, including the rate of successful attachment and replication, using models [17,18]. Phage replication also depends on the growth of bacterial cells. When they are in the log phase, replication occurs exponentially due to increased metabolic activity. Phages take advantage of this phase for replication as well. In the stationary phase, phage replication also decreases [19].

The dissemination of genes among bacteriophages occurs in three distinct forms: generalized, specialized, and lateral transduction.

### 2.1. Generalized Transduction

**Figure 1:**
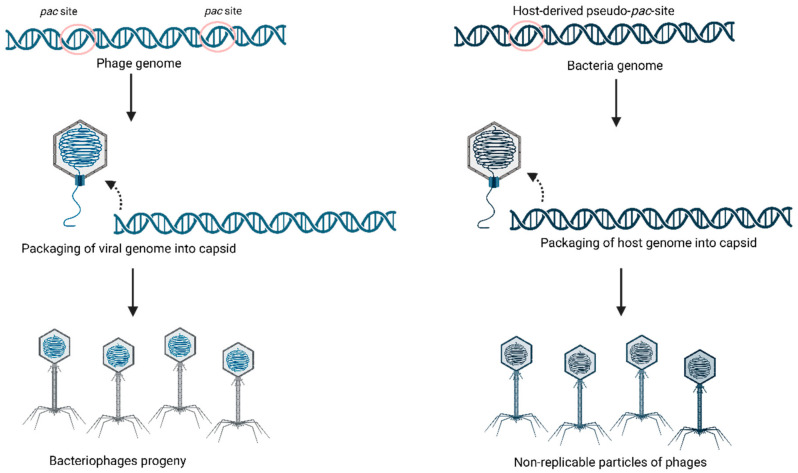
Generalized transduction: the mechanism by which bacteriophages transfer DNA fragments from one bacterial cell to another.

The generalized transduction is a transduction by which phages package DNA from the host into capsids. The host DNA is incorporated and recognized by mistake. The enzymes terminases are responsible for identifying *pac*-type regions indicating phage genome, but the presence of pseudo *pac*-type can lead to an error, making the terminase package the wrong amount of DNA from the host, as presented in Figure 1.

### 2.2. Specialized Transduction

**Figure 2:**
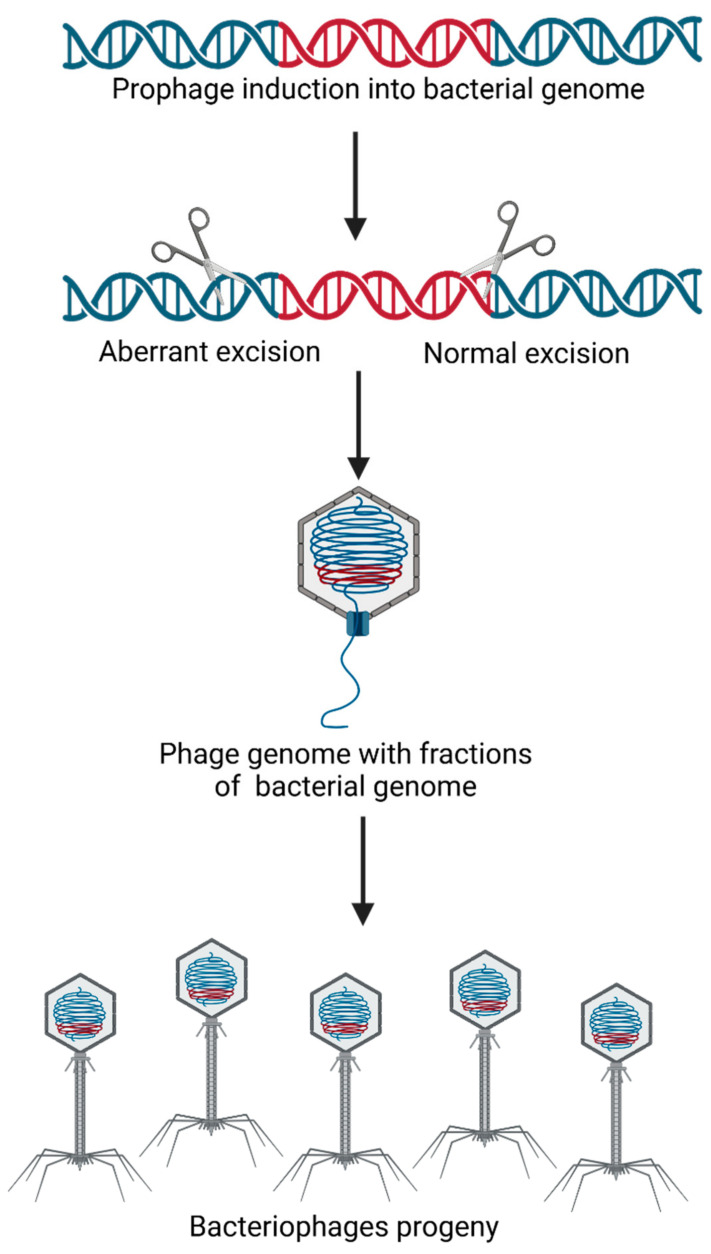
A process in which bacteriophages transfer specific DNA fragments from the bacterial genome, near to prophage integration site.

Specialized transduction was first discovered in coliphage λ being the second transduction mechanism identified. Different from generalized transduction, which can package any segment of bacterial DNA, specialized transduction can transfer specific groups of genes. During this process, phage particles are built with hybrid molecules consisting of both viral and host bacterial DNA. The mechanism was characterized in coliphage λ, where aberrant excision of the viral genome frequently includes adjacent host DNA segments. If a *cos* site is retained, the hybrid DNA is packaged into capsids by the *cos*-type packaging machinery and subsequently transferred to new host cells, as presented in Figure 2. Nonetheless, this transduction is relatively rare, and the transfer is limited to specific DNA fragments, resulting in a small volume of gene transfer [20].

### 2.3. Lateral Transduction

It is known that generalized and specialized transduction occur due to errors in the process of incorporating host DNA with or instead of the phage genome. In contrast, lateral transduction, a new mechanism discovered in phages of *Staphylococcus aureus*, is known as the third mechanism. This process can result in transference at frequencies at least 1000 times higher than those observed in other transduction methods.

Normally, prophages excise and circularize early during lysogenic induction, following the standard excision-replication-packaging process. This step keeps the viral genome intact before replication and packaging. However, it was possible to identify an alternative sequence in the phages of *S. aureus*. The prophage excises later in the lytic cycle, which can alter the excision-replication-packaging order, creating a delay in excision. This delay directly affects the gene transfer because the phage enzyme terminase begins DNA packaging in situ while the prophage remains attached to the host chromosome.

**Figure 3:**
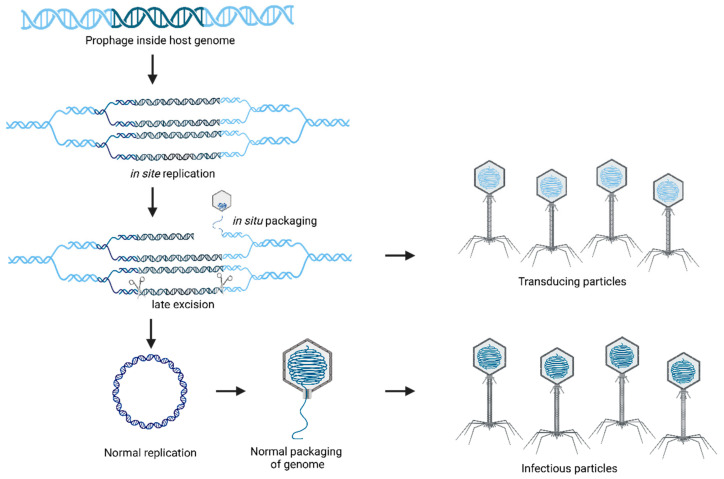
Phage particles that mistakenly package bacterial DNA instead of viral DNA, playing a crucial role in horizontal gene transfer during transduction.

DNA packaging starts at the *pac* site and proceeds directly into the host chromosome. Consequently, several hundred kilobases of bacterial DNA can be packaged into phage heads and transferred at higher frequencies than other gene transfer mechanisms. To protect the viral genome from cleavage during the packaging process, the phage induces theta replication in the bacterial chromosome, allowing multiple integrated genomes to mature and be packaged simultaneously. This mechanism has been described as a “hypermobile platform” for gene transfer, as presented in Figure 3 [21].

## 3. Natural Reservoirs of ARGs and Influence on Phage Transference

Since the beginning of antimicrobial compound manipulation for human and animal production, the emergence of resistance genes has been influenced. Various environments, such as terrestrial and aquatic ecosystems, have been identified as reservoirs for these genes [22]. However, recent analyses of ARGs in areas unexplored by humans oppose this idea. Certain levels of antibiotic-resistant genes were identified in areas where human activities are low, such as aquatic and terrestrial extreme environments, previously considered free of ARGs [19,23,24,25].

This observation has led to the resistome hypothesis, which suggests that some ARGs associated with clinical pathogens originated in antibiotic-producing environmental bacteria and were later transferred to pathogens through HGT, such as bacteriophage-mediated transduction [26,27].

The concept of the resistome considers that bacteria with the capability of producing antibiotic compounds, such as those from the genus *Streptomyces*, which are prevalent in soils worldwide, have developed resistance genes to protect themselves from their own metabolic products with antibiotic capabilities—a phenomenon described by the “producer hypothesis”. These organisms often exhibit multidrug resistance, either due to their ability to produce multiple antibiotics or because of their evolution alongside other antibiotic-producing organisms in soil ecosystems. The environmental resistome is thus recognized as a significant source of resistance genes, many of which are believed to have entered clinical pathogens over time.

Aminov and collaborators (2011) establish links among groups of ARGs from environmental to clinical [28]. ARGs associated with extended-spectrum β-lactamase *CTX-M* and quinolone resistance were identified in environmental species such as *Kluyvera* and in *Klebsiella pneumoniae* isolated from the environment [29,30]. A study of 30,000-year-old Beringian permafrost identified genes to β-lactams, tetracyclines, and glycopeptides. These genes suggest their presence in the environment years before the manipulation and distribution of these antibiotics in our society [31].

Although this research established that resistance to natural antibiotics predates human intervention, a temporal study conducted in agricultural soils from the Netherlands observed a significant rise in ARG prevalence from the pre-antibiotic era (1940s) to modern times [32]. In contrast, earlier investigations showed that plasmids from pathogenic bacteria were isolated before antibiotics were widely used and lacked resistance genes [33]. Resistance to natural antibiotics is an ancient phenomenon, although the distribution and production of antimicrobials compounds in large scale may have played a role in its spread and transfer worldwide. Some unexplored ecological niches could reveal bacteriophages facilitating the transfer of ARGs from environmental microbes to humans and animals [27,34,35].

One Health is a concept that links environmental, animal, and human health. Certain practices, like using contaminated water for irrigation or applying biosolids and manure as fertilizers, can promote the spread of antibiotic resistance genes (ARGs) across various environments. As a result, these contaminants can make their way through the food chain, potentially impacting public health.

Poor sanitation conditions can contribute to the spread of pathogens, especially through contaminated water and food, risking human and animal health. These environments become hotspots for bacterial pathogens from different sources [36]. Antibiotics have been used in our society for treatments, prevention of disease, and as growth promoters in animals. The administration of antibiotics may have selected antibiotic resistance genes, which can return to the environment through feces. Animal manure, for example, is used as a crop fertilizer, which can potentially introduce residual antibiotics into the soil [37,38]. Considering that, public policies have been implemented to mitigate ARG dissemination. In the year 2006, the European Union banned the use of antibiotics as growth promoters in animal feed. In June 2019, new veterinary regulations extended these restrictions, introducing a complete ban on specific antibiotics [39]. During the year 2017, the U.S. Food and Drug Administration (FDA) prohibited the administration of antimicrobials to livestock as growth promoters [40].

Bacterial pathogens which contaminate food are known as foodborne. They can contaminate food in different stages–farming, animal slaughter, food handling, industry and preparation [41]. The bacteria Campylobacter and non-typhoidal Salmonella are common pathogens, related to social and economic conditions. In high-income countries, Campylobacter is prevalent, while in lower-income countries *Salmonella typhi*, *Vibrio cholerae* and *Escherichia coli* strains are prevalent [42].

## 4. Dissemination of ARGs by Lysogenic Phages in the Food Chain

The correlation between ARGs and lysogenic phages has been studied, with some studies isolating phages and fractions of phages from different matrices. Colomer-Lluch et al. (2011) demonstrated that phage fractions can carry ARGs to bacteria in cattle, pigs, and poultry; Al-Mustapha (2023) identified bacteriophages containing open reading frames capable of transmitting these genes to bacterial hosts [43,44].

Yang (2019) identified genes *mcr-1*, *aac(6′)-Ib-cr*, *blaCTX-M*, *ermB*, *floR*, *sul1*, *tetM*, located in the phage genome [45,46,47]. In Vienna, Austria, Shousha (2015) analyzed coliphages in chicken meat products and found that 22 out of 50 samples contained phages capable of transmitting resistance to antibiotics such as kanamycin, chloramphenicol, ampicillin, and tetracycline. Approximately 24.7% of the coliphages could transfer ARGs to *E. coli* ATCC 13706, a strain without any resistance genes. After replication of different phages, *E. coli* with resistance genes was obtained through the process of lysogeny [48].

### 4.1. ARGs in Animal Products and Their Persistence in the Food Chain

Anand and collaborators (2016) isolated bacteriophages from different environments—soil, water, and sewage. They identified the genes *tet(A)* (12.7%), *intI1* (10.9%), *intI2* (10.9%), *intI3* (9.1%), *tetW* (9.1%), *blaOXA-2* (3.6%), and *blaTEM* (30.9%) located in phage genomes [49]. Wang and collaborators (2018) investigated bacteriophages from pig feces, finding *sul1*, *blaTEM*, and *ermB* in 100% of the samples. Other detected genes included *qnrA* (96.2%), *qnrS* (92.1%), *fexA* (83.6%), *floR* (82%), *aac(6′)-Ib-cr* (46%), *cfr* (38.6%), *tetM* (31.2%), and *blaCTX-M-1* (21.2%) [50].

In China, Yang (2020) [46] also identified bacteriophage DNA from 30 farm chicken fecal samples. He identified 12 resistance genes: (*aac(6′)-Ib-cr*, *aph(3′)-IIIa*, *blaCTX-M*, *ermB*, *ermF*, *floR*, *mcr-1*, *qnrS*, *sul1*, *sul2*, *vanA*, and *tetM*), along with the *intI1* gene. The most frequent genes were *sul1* (77%), *tetM* (63%), and *aac(6′)-Ib-cr* (53%). These studies suggest that poultry feces are possible reservoirs of ARGs, which can be influenced by the administration of antimicrobials [51,52].

### 4.2. Comparative Analyses in Poultry

Different matrices can show a variation of levels of ARGs identified. Depending on the matrix type, animal production, and administration of antibiotics, these genes can vary. For example, by comparing broilers, broiler breeders, slaughterhouses, and laying hens, it was possible to detect different genes and their quantities in each group. The matrix with the lowest ARGs was laying hens, possibly be due to lower administration of antimicrobial in these animals [53,54,55].

Beyond resistance genes, there are toxins that can be produced by certain strains, for example Shiga toxin, produced by *E. coli* serotype O157:H7. *E. coli* that are capable of producing Shiga toxin are called Shiga toxin-producing *E. coli*—STEC. Those strains in food products pose a risk to food consumption due to the chance of contamination. The first outbreak identified to be caused by Shiga toxin was in 1982, in Oregon and Michigan, in a fast-food chain [56].

Even after the characterization of these strains, STEC remained a concern in food production. Strains isolated from clinical and environmental settings presented resistance to antibiotics such as streptomycin, sulfonamides, and tetracyclines [57,58]. Also, isolates of toxin producers identified in Ireland exhibit multidrug resistance to 10 different antimicrobials [59].

Phages can influence the adaptability of toxin-producer strains. For example, Stx-phages isolated from *E. coli* O157:H7 have successfully transferred tetracycline resistance genes to laboratory strains of *E. coli* [60]. Also, recombinant Stx-phages engineered to carry chloramphenicol and tetracycline resistance genes produced transductants in susceptible strains like DH5α and C600 [61].

The influence of phages extends beyond toxin producers. Brüssow and collaborators (2004) reviewed phage transference of virulence factors across different bacterial species. For example, in *Vibrio cholerae*, the acquisition of two fitness factors—the toxin coregulated pilus (TCP) and cholera toxin (CT), both encoded by phages—enhances its virulence [13].

Gómez-Gómez (2019) identified the genes: *sul1*, *armA*, *mecA*, *blaOXA-48*, *blaVIM*, *qnrS*, *blaCTX-M-1*, *blaCTX-M-9*, *blaTEM*, and *qnrA* in samples of pork, beef, chicken feces, and ham. The two most prevalent genes were *sul1* and *blaTEM* [62].

In another study, Blanco-Picazo (2022a) evaluated a virome using meat (pork, veal, and poultry), fish (shellfish, farmed, Mediterranean frozen, and Atlantic), and vegetables (cucumber, lettuce, and spinach) as subtracts, confirming that ARGs found in phage particles originated from bacterial hosts. Notably, veal samples showed the highest ARG diversity despite minimal bacterial contamination, underscoring the limitations of using bacterial indicators to predict viral contaminants [63,64,65].

### 4.3. Phages in Dairy Products

Dairy products are matrices where phages can reside. Phages can be detected in dairy environments, persisting even after the process of pasteurization. Contamination can occur from raw materials or starter cultures of bacteria during production [37]. Chopin (2001) and Madera (2004), for example, identified phages in horchata and from starter cultures used in production [66,67]. In another study, Blanco-Picazo (2022b) identified phages in dairy products, although these phages could not be replicated in *E. coli* species [68]. Contamination with phages can happen during the production processes of dairy products. Milk can accumulate bacteria from different sources, as well as phage virions [69]. Additionally, phages can be induced from prophages present in the milk microbiota, occasionally disrupting fermentation processes [70]. The replication by bacteriophages of lactic acid bacteria can interfere with fermentation, decreasing production and financial gains [71].

Kerek and collaborators identified ARGs in raw milk and dairy products using next-generation sequencing to monitor changes in the ARG pool from farm to consumer. Beyond that, they identified the composition of each pool with predominance of groups of bacteria. In raw milk, the microbiota was predominantly composed of Firmicutes (41%) and Proteobacteria (58%). This composition was different in fresh cheese (93% Firmicutes, 7% Proteobacteria) and matured cheese (79% Firmicutes, 21% Proteobacteria). In total, 112 antimicrobial resistance genes were identified, including extended-spectrum β-lactamase genes [72].

Madera et al. (2004) identified phages in dairy products [67]. They found that 9.2% of raw milk samples were contaminated with phages, predominantly c2-like phages. The species contaminated with phages was *Lactococcus lactis*. Moineau and Lévesque (2004) compiled data from several years, correlating phage infections in the fermentations process in food products, while most studies were not specifically focused on the transmission of ARGs [73].

Despite the efforts to identify studies where the same resistance genes are found in both bacteria and bacteriophages isolated from food matrices, there are relatively few articles focusing on this topic [46,63,68,74,75]. This demonstrates the necessity of further exploration to understand the simultaneous presence of resistance genes in both bacterial and phage genomes within food matrices and food systems productions.

## 5. Farm to Fork

### 5.1. Agricultural Practices

Farm-to-fork includes a long chain of food production, and in its many steps there are several gaps which represent opportunities for contamination by antibiotic-resistant microorganisms. Farms can be contaminated by sewage, manure, agricultural waste, and practices like soil treatment with dairy manure, urban wastewater sludge, or reuse of water [76,77,78,79].

Sub-doses of antibiotics administered in livestock can select resistant bacteria, facilitating their proliferation within animal guts, and leading to their excretion in feces [80]. Around 80% of all antibiotics can persist as active compounds in manure as residues [81,82]. When applied as fertilizer, this manure introduces resistant bacteria and residual antibiotics into soils. There are some characteristics that can influence the persistence of residual antimicrobial compounds, such as the degradation rates of molecules, hydrophobicity, and sorption dynamics [83,84].

In soil utilized in agriculture, some studies demonstrated the correlation between the use of organic fertilizers and manure with levels of ARGs (e.g., *sul1*, *tet(W)*, *blaCTX-M*) [83,85]. Ross and Topp (2015) correlate the use of manure and biosolids with the prevalence of ARGs [75]. Considering the depth of soil that receives swine manure, ARGs are more abundant at greater depths than in the surface layer [84,86]. Analysis of slaughterhouses in Spain and Tunisia identified resistance genes *sul1*, *blaTEM*, *blaCTX-M-9*, *blaCTX-M-1*, *mecA*, and *armA* in phage and bacterial fractions. The gene *blaCTX-M-9* was more prevalent than *blaCTX-M-1* in the samples [43].

### 5.2. Food Processing and Storage Environments

In facilities, various disinfectants are applied to eliminate bacteria. However, sub-lethal doses of these disinfectants can promote the development of resistant microbial strains, leading to their persistence within the processing environments. Furthermore, ARGs can be acquired by neighboring bacteria through transduction events mediated by bacteriophages. Haaber (2016) demonstrated that after the infection of phages in *Staphylococcus aureus* strains, subpopulations of *S. aureus* contain prophages. This subpopulation with prophages can acquire genes from susceptible strains, a process called “auto-transduction” [87].

In contrast, bacteriophages have reemerged as a promising alternative for combatting antibiotic-resistant bacteria. Recent studies have demonstrated the effects of phage predation on the spatial organization of microbial populations. Phage predation can increase the spread of plasmid-encoded antibiotic resistance during surface microbial growth. This mechanism involves faster-growing cells from the biomass periphery to its interior, where cells are packed more closely together [88].

### 5.3. Retail and Consumer-Level Interactions

As discussed, vegetables cultivated in opens fields are susceptible to contamination from different sources, such as soil, fertilizers, and irrigation water. Vegetables that are consumed raw can retain microorganisms on their surface. In this way, a study confirmed ARGs within phage DNA fractions from soil and vegetables. Among the ARGs identified in lettuce and soil β-lactamase was predominantly found [74]. Another study identified 60 phage plasmids containing 184 ARGs conferring resistance to broad-spectrum cephalosporins, carbapenems, aminoglycosides, fluoroquinolones, and colistin [89].

The literature also reports on the presence of phage particles carrying ARGs in products such as meat, pork, beef, minced chicken, ham, and mortadella. The sulfonamide resistance gene (*sul1*) was most abundant, followed by β-lactamase genes. It was possible to detect ARGs in the phage DNA fraction of chicken fecal samples, confirming the presence of genes in excretions [62]. Once prophages integrated into bacteria are released, they can transfer ARGs to other bacteria; studies confirm the transference to *E. coli* O157:H7 and *Salmonella* spp. [90,91].

#### 5.3.1. Mechanisms by Which Lysogenic Phages Spread ARGs in the Food Chain

Lysogenic phages are also capable of serving as agents in the dissemination of antibiotic resistance genes (ARGs), propagating through the food chain. This is an extreme example of the cross-boundary transfer of ARGs in microbial consortia associated with plants. In other scenarios, such as in livestock rearing, where pigs, chickens, cattle, and fish are commonly grown, the farm environment is very conducive to bacteria and phage interactions, thus enhancing the proliferation of lysogenic phages [92].

The CTX phage does not kill but rather alters the physiology of the cholera-producing microorganism and enables it to produce cholera toxin, resulting in severe dehydration in a cholera infested area [93]. Pathogenic bacteria that are present in bacteria-contaminated food or water may produce the toxin in greater quantities. For example, in *E. coli*, the production of Shiga toxin is assisted by bacteriophages of lambdoid origin. When *E. coli* is lysogenic, the new phages enter a latent state, allowing them to integrate into the *E. coli* chromosome and increasing the amount of non-O157 strains that can change lysogenically [34].

Cholera caused by *V. cholerae* is also due to CTX phages carrying the cholera toxin genes, *ctx*A and *ctx*B. These phages integrate into the genome of the bacterial cell and may be found on either of the two V. cholerae chromosomes or located in tandem on the larger one [94]. The CTX phage genome, approximately 6.9 kb in size, contains two significant regions: the core region, which consists of toxin genes and structural proteins that form the viral coat, and the RS2 region, responsible for replication, regulation, and integration.

The soil in agricultural ecosystems also harbors phages with ARGs and is also one of the causes of the horizontal transfer of resistance genes to crops. The use of is another cause of the horizontal transfer of resistance genes to crops. Cattle farming also increases the trading of ARGs among animal gut microbes, resulting in resistant strains that have a high probability of infecting meat on the slaughter block. These resistant bacteria can be transferred to human food chains via contaminated fruit or meat [92].

Keen et al. originally referred to such phages as “superspreader” phages. This occurs when phages release intact and transformable bacterial DNA from cell lysis so that even the plasmid-mediated HGT in bacteria that are not within the host range is accessible to the phages. In order to function in this position, SUSP1 and SUSP2 phages discharge such plasmids as pOAR31 and pπγ efficiently, the latter being vectors of the environment with functions related to resistance determinants and the transfer of virulence. Superspreaders are another example that facilitated the transfer of kanamycin resistance from *E. coli* to a *Bacillus* soil isolate, which would not occur directly with SUSP2 infection. This demonstrates the efficacy of superspreader phages in transcending host-range restrictions and widening the ecological niche of resistance determinants [95].

The activity of superspreader phages may be more associated with some inherent properties, such as naturally effective bacterial population, environmental stability of plasmid DNA, and microbial host range sustaining the disseminating plasmids. Contribution of one superspreader phage to the food web is unknown, but the ability of superspreader phages to spread ARGs into various microbial communities suggests that perhaps additional effort would be needed.

#### 5.3.2. Contamination During Food Processing

Transduction is a form of phage–bacteria interaction and thus cannot be prevented. But the presence of some antibiotics that may be used within food production systems and that are being stored in animal and human tissues can also increase the level of transduction activity to create new resistances. In the food web, the transduction of the food process network, or in the intestines of animals and humans upon consuming food, may initiate gene transfer by the gut microbiota [96].

Food processing involves numerous processes that are contaminated with phage-infected ARGs or ARB carriers, such as dirty cleaning, contaminated water, and mechanical processing in the forms of grinding, cutting, and mixing that can spread ARGs as well as expose surfaces to phage–bacteria interaction. Throughout the above processes, phage-lysed bacteria can seep DNA into the food matrix where it becomes entrapped by phages of lysogenic origin. Apart from this, other equipment surfaces utilized in the aforementioned processes are biofilm-permissive surfaces and, therefore, HGT-permissive [97].

Bacterial fermentation processes conducted repeatedly in the dairy, meat, and vegetable product industries are also causative, i.e., lactic acid bacteria’s susceptibility to contamination by lysogenic phages, particularly in the starter cultures used in the majority of these processes. Such an operation will disrupt fermentation process and transfer ARGs to microbial communities [70,71]. Antimicrobial residue presence in raw material may also potentially impose selection pressure enabling the increase in frequency of transduction event.

Lastly, storage and packaging conditions themselves may be the route of contamination, if not managed. Phages would remain viable and infect bacterial populations following heat treatment or contact with phage-contaminated pack materials. Chill storage might inhibit bacterial growth, but the phages would be stable enough at this temperature to facilitate long-term ARG transfer.

Rodríguez-Rubio et al. (2020) published a study wherein ARG packaging was performed by MCP phages in *E. coli* strains [98]. Different phages, i.e., *Podovirus Stx* phages and *Myovirus Cdt* phages, were used to inoculate the strains. The results were complemented with phages that can package and transfer ARGs from MCPs, suggesting their likely function in enhancing ARG transmission in food processing.

## 6. Uncertainties and Challenges in Understanding Lateral Transduction

Lateral transduction remains underexplored and is yet to be fully understood in its complete potential. As of now, this has been known only for a few species of bacteria, such as *Staphylococcus aureus*, *Salmonella enterica*, and *Enterococcus faecalis*. Currently, whether such a phenomenon is limited only to these bacteria or could possibly exist more variably across various bacterial taxa [99,100] remains to be discovered. The system is prophage dependent, and prophages prevent excision from the bacterial chromosome, enabling packaging to begin at the pac site and incorporate proximal chromosomal regions tens to hundreds of kilobases in length. Such site specificity proximal to the site implies that lateral transduction may have characteristic ranges and boundaries [101].

Current evidence for phage-mediated horizontal gene transfer relies mainly on culture-based methods that, in principle, eliminate contact with nonculturable bacteria. Metagenomics and allied technologies have enlightened us on this aspect, but are subject to caveats such as: (i) contamination during the sample processing step by bacterial DNA, which magnifies phage-contributed ARGs; and (ii) the need for phage nucleic acid purity, free from bacterial contaminants, regardless of purification treatments such as density gradient centrifugation or cesium chloride [102].

New approaches have been developed to address such challenges. Transductomics, for example, facilitates the direct analysis of phage-transferred DNA for unveiling of the horizontal gene transfer dynamics data. With an approach like this, such work has been able to detect functional resistance genes, e.g., β-lactamase genes, in water and assigned phages as ARG carriers. Interestingly, this study shows that ARGs are more prevalent in free phage particles than prophages in bacterial genomes [103]. In light of this, the general implication of phages in the dissemination of ARGs is disputed as evidence contradicts each other depending on habitat and microbial assemblage.

With that proviso, examples of lateral transduction need to be moderated. Although the process has a high capacity to mobilize DNA, its frequency and ecological impact have yet to be comprehensively investigated. The integration of genomic, metagenomic, and functional analysis in scientific research will be important for understanding the role of phages in disseminating antibiotic resistance and distinguishing their role from other mobile genetic units, such as plasmids [34].

## 7. Conclusions

Bacteriophages play a dual role in microbial ecosystems, acting both as agents of genetic innovation and as potential threats to food safety. Despite advances in food safety aimed at reducing contamination, inhibiting bacterial persistence in processing facilities, and minimizing the presence of ARGs and toxins in foodborne pathogens, critical knowledge gaps remain. The prevalence, dynamics, and ecological drivers of phage-mediated gene transfer in complex food production environments are still poorly explored. The lateral transduction and its role on the acquisition of ARGs, together with the presence of ARGs in other groups of bacteria in the same food-related environments, pose a risk to food safety. In addition, the unexplored relationship between bacteriophages in industry, animal production, and dairy products highlights the limited current knowledge in understanding these dynamics. These gaps highlight the need for comprehensive research to better understand the interactions between bacteriophages and bacterial hosts throughout the food chain.

The use of different approaches to tracking mobile genetic elements in different matrices can be efficient to mitigate their spread. Whole genome analysis, metagenomics, viromics, and transductomics can be useful for providing insights into HGT, pathogenic islands, and lateral transduction. Reducing the cost of these technologies and expanding their use, as well as using specialized techniques for rapid identification, could provide further insights into how transduction and movement of mobile genetic elements occur in different matrices in the coming years. By integrating advanced genomic tools and interdisciplinary collaboration, advances in public health and the food supply can be achieved.

## 8. Future Perspectives and Research Directions

While lateral transduction has been identified as a highly efficient mechanism for transferring large amounts of genetic material, other emerging pathways, such as those mediated by gene transfer agents (GTAs) and phage-inducible chromosomal islands (PICIs), also requires attention.

GTAs resemble phages morphologically and functionally, but phages differ in that they are not capable of packaging their genetic material. GTAs encapsulate a random piece of the host cell genome (typically 4 to 14 kb) for horizontal transmission. Phages are distinct in the sense that the expression of the gene cluster is regulated by processes of the host cell, generally quorum-sensing pathways, rather than phage repression systems. Deformed virus PBSX of *Bacillus subtilis* assembles virus-like particles (VLPs) through a mechanism related to GTA action. These VLPs carry random 13 kb blocks of chromosomal DNA but do not form detectable transducers. They work as bacteriocins with growth-inhibitory activity on non-carrying cells and put selective pressure on maintaining the PBSX element within populations [104,105]. Although GTAs are poorly understood in the food web environment, they have a critical role in promoting microbial fitness in agroecosystems and food-producing systems and warrant increased investigation.

PICIs are known as another class of mobile genetic elements that can be transported by HGT. PICIs, such as SaPIs in *Staphylococcus aureus*, can be found in both Gram-negative and Gram-positive bacteria. They are integrated and remain dormant until stress conditions or helper phages act. After induction, PICIs are excised from host genome and included in packaging of phages. Those islands present in PICIs can be associated with diverse groups of genes, including virulence, toxin, and antimicrobial resistance [54,96]. PICIs are a serious threat to microbial control in food systems, given their ability to exploit phage machinery alongside their ability to contribute to ARGs dissemination in foodborne pathogens.

The flexibility provided by GTAs and PICIs can enable microbial populations to coexist and thrive under selective pressure, i.e., that of antibiotic use in agriculture or disinfection schemes in crops of food plants. The combination of metagenomics, virome sequencing, and transductomics may be useful in elucidating interactions among HGT transferred by phages. Furthermore, expanding research to field environments—e.g., agricultural field soil, irrigation systems, and food-processing plants—will result in applied knowledge about phages’ ecological dynamics and HGT [101,102].

Regulatory responses and technological advances must keep up with this growing body of evidence. ARG dissemination must be addressed by policy to maintain the risks at a minimum. Moreover, bacteriophage production as biocontrol products may serve as a viable substitute for foodborne pathogen control and antibiotic reduction.

## Data Availability

No new data were created or analyzed in this study. Data sharing is not applicable to this article.

## Abbreviations

The following abbreviations are used in this manuscript:

ARG	Antibiotic genes resistance
HGT	Horizontal gene transfer
GTA	Gene transfer agent
PICI	Phage-inducible chromosomal islands
VLP	Virus like particle
MCP	Multicopy plasmids
ARM	Antimicrobial resistance
STEC	Shiga toxin-producing *E. coli*
TCP	Toxin-coregulated pilus
TC	Cholera toxin
VPI	*V. cholerae* pathogenicity island
